# Latest update on chemokine receptors as therapeutic targets

**DOI:** 10.1042/BST20201114

**Published:** 2021-06-01

**Authors:** Wing Yee Lai, Anja Mueller

**Affiliations:** School of Pharmacy, University of East Anglia, Norwich, U.K.

**Keywords:** chemokines, signalling, therapeutics

## Abstract

The chemokine system plays a fundamental role in a diverse range of physiological processes, such as homeostasis and immune responses. Dysregulation in the chemokine system has been linked to inflammatory diseases and cancer, which renders chemokine receptors to be considered as therapeutic targets. In the past two decades, around 45 drugs targeting chemokine receptors have been developed, yet only three are clinically approved. The challenging factors include the limited understanding of aberrant chemokine signalling in malignant diseases, high redundancy of the chemokine system, differences between cell types and non-specific binding of the chemokine receptor antagonists due to the broad ligand-binding pockets. In recent years, emerging studies attempt to characterise the chemokine ligand–receptor interactions and the downstream signalling protein–protein interactions, aiming to fine tuning to the promiscuous interplay of the chemokine system for the development of precision medicine. This review will outline the updates on the mechanistic insights in the chemokine system and propose some potential strategies in the future development of targeted therapy.

## Introduction

Chemokine signalling was initially identified to be a key coordinator in leukocyte trafficking in immune responses and inflammation [1,2]. In the past two decades, emerging evidence shows that dysregulation of the chemokine system is implicated in the pathogenesis of cancer [3]. Different ligand–receptor pairs have been demonstrated to be involved in distinct pro- and anti-tumour functions in cancer, such as angiogenesis, proliferation, differentiation, immune evasion and metastasis [4]. The overexpression profile of chemokine receptors is different in a variety of cancers, such as breast cancer, pancreatic cancer and lung cancer [5,6]. In light of this, chemokine receptors have become potential targets for cancer targeted therapy.

In the past two decades, numerous attempts have been developing drugs targeting chemokine receptors, and yet only 3 out of 45 are clinical approved [7]: Maraviroc, a CCR5 allosteric antagonist for anti-HIV [8]; Plerixafor, a CXCR4 antagonist for non-Hodgkin's lymphoma and multiple myeloma [9]; Mogamulizumab, an anti-CXCR4 monoclonal antibody for T cell leukaemia and cutaneous T cell lymphoma [10]. Why is it so challenging to employ chemokine receptors as therapeutic targets? First, the chemokine system is so complex [11–13]. A thorough understanding of the redundancy of chemokine receptor-ligand binding remains obscure. Secondly, chemokine signalling plays a key role in pathogenesis while it is also important in physiological processes [2,3,12,13]. Moreover, the broad, open features of chemokine receptors add difficulties to designing chemokine receptor antagonists without any non-specific binding [14].

In recent years, considerable efforts have been made to improve the understanding of the complexity of the chemokine system. Thanks to the latest techniques, fluorescence (FRET) and bioluminescence (BRET) resonance energy transfer have been broadly used for characterising the chemokine ligand–receptor interactions and protein–protein interactions in live cells [15,16]. Recent research trend aims to modulate the intricate signalling pathway in the chemokine system which has been a step forward in the development of precision medicine. This review will summarise recent discoveries regarding biased signalling in the chemokine system and illustrate how the proposed mechanisms potentially transform into the strategies in the discovery and development of targeted therapy.

## Background on the chemokine signalling system

The chemokine receptors belong to the class A of G protein-coupled receptor (GPCR) superfamily. GPCRs are characterised as a seven-transmembrane domain with a flexible extracellular N-terminus connected to the intracellular C-terminus through three extracellular (ECL) and three intracellular (ICL) loops [17,18]. To date, 19 canonical chemokine receptors, including CC, CXC, CX3C and XC subfamilies, and four atypical chemokine receptors (ACKRs) have been characterised in humans [17,19]. The structure of chemokine receptors is highly conserved in the presence of a DRYLAIV motif within the second ICL, except for the ACKRs [19,20].

**Figure 1.**
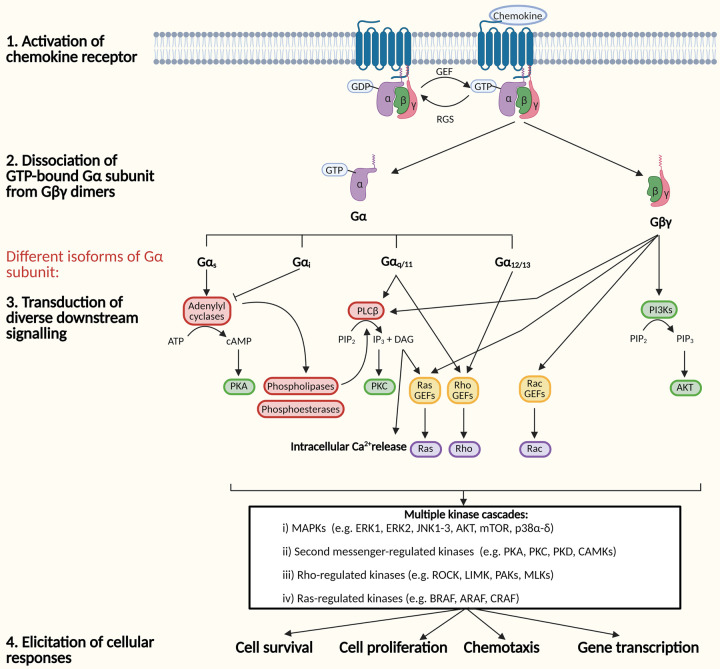
G protein-dependent signalling. Schematic diagram of the distinct signalling pathways upon chemokine receptor activation through G proteins (Abbreviations: ATP, adenosine triphosphate; cAMP, cyclic adenosine monophosphate; DAG, diacylglycerol; GDP, guanosine diphosphate; GEF, guanine nucleotide exchange factor; GTP, guanosine triphosphate; IP_3_, inositol trisphosphate; MAPKs, mitogen-activated protein kinases; PI3Ks, phosphoinositide 3-kinases; PIP_2_, phosphatidylinositol (4,5)-bisphosphate; PIP_3_, phosphatidylinositol (3,4,5)-trisphosphate; PKA, protein kinase A; PKC, protein kinase C).

In the inactivated state, chemokine receptors are coupled to a heterotrimeric GTP-binding protein (G protein) comprising of α, β and γ subunits [21,22]. Upon chemokine ligand binding, the activation signal promotes the exchange of GDP for a GTP at the Gα subunit, leading to the dissociation of the GTP-bound Gα subunit from Gβγ dimers. Gα and Gβγ subunits separate from the receptor to transduce downstream signalling accordingly [22]. Gα proteins can be subdivided into four isoforms: Gα_s_, Gα_i_, Gα_q/11_ and G_12/13_, which possess differential functionality. The activated chemokine receptor is able to induce the activation of one or multiple Gα and Gβγ proteins at the same time [22]. Generally, the differential G protein signalling involves intracellular calcium mobilisation, stimulation or inhibition of cAMP production and activation of second messengers, such as Ras, Rho and Rac. These second messengers in turn stimulate multiple kinase cascades regulating cellular functions, contributing to chemotaxis, gene transcription, cell survival and proliferation (detailed in Figure 1) [23].

**Figure 2.**
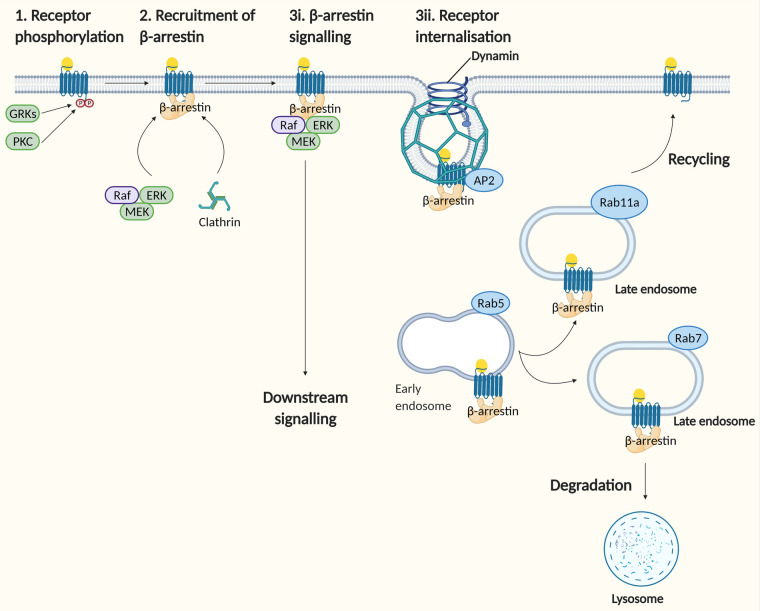
G protein-independent signalling through β-arrestin recruitment. Schematic diagram of the downstream signalling pathway and receptor endocytic sorting upon chemokine receptor activation through β-arrestin recruitment (Abbreviations: AP2, adaptor protein 2; ERK, extracellular-signal-regulated kinase; GRK, G protein-coupled receptor kinase).

In addition to G protein-dependent signalling, some chemokine receptors initiate signalling independent of G proteins. The critical mediator in this pathway is a family of β-arrestin proteins, which serves as binding scaffolds at the cytosolic face of the receptor for the recruitment of the endocytic machinery [24], as well as kinases, to elicit intracellular signalling [25,26] (detailed in Figure 2).

## Chemokine receptor conformational selection model in receptor activation

Current studies have established a chemokine receptor conformational selection model [18,19,27] as a structural basis in chemokine receptor activation to replace the conventional two-step model [18]. In the initial step of receptor recognition, the N-loop of chemokine binds the N-terminus of the chemokine receptor, known as chemokine recognition site 1 (CRS1) [18,27]. Within the ligand–receptor interactions, it involves dynamic conformational changes in the chemokine ligand with multiple binding sites that facilitates the activation of the receptor [27]. To elucidate the diversity of chemokine signalling from different ligand–receptor pairs, the post-translationally modified residues at the N-terminus of the chemokine receptor are the crucial components for contacting their cognate ligands to initiate biased signalling. For example, sulfation at three tyrosine residues for CXCL12/CXCR4 pair [28] and polysialylation for CCL21/CCR7 pair [29]. In the second step of receptor activation, the N-terminus of chemokine interacts with the receptor transmembrane domains named as CRS2 [18,19,27]. Numerous literatures show that the cysteine motif in chemokine is the critical component in receptor activation [30–32]. A recent study yields an insight into some other motifs of the N-terminal residues in chemokine that potentially modulates cross-talk between CRS1 and CRS2 of the chemokine receptor. In particular, the GP motif of the ELR residues in CXCL8 is an important regulator in mediating multiple receptor signalling pathways [27]. In summary, a specific N-loop and specific motif in the N-terminus of chemokine are the key determinants for canonical chemokine receptor selectivity and activity.

Though there is an exceptional case for ACKR3. In ACKR3, a specific N-loop of chemokine is dispensable for receptor activity and alterations in N-terminal residues cause minimal effects in receptor potency [33]. Whether this exceptional observation applies to other ACKRs remains to be investigated.

## Biased signalling in the chemokine system

**Figure 3.**
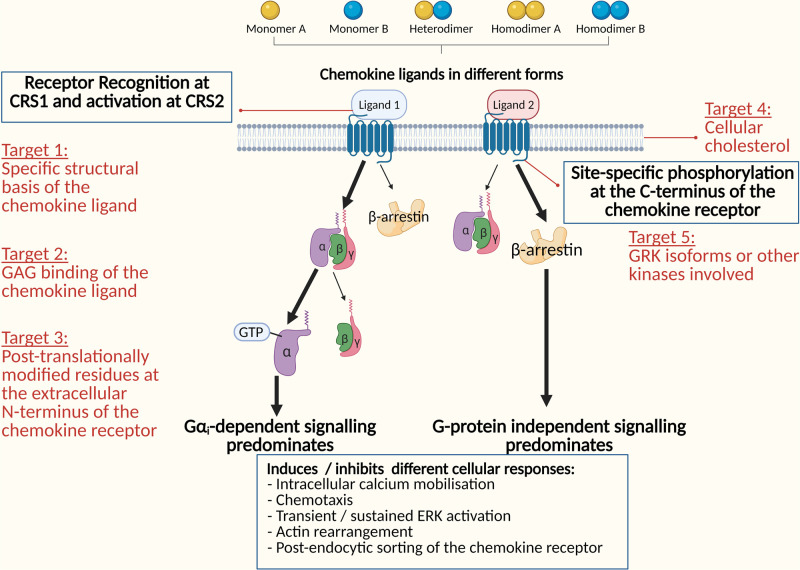
Biased signalling in the chemokine system. Schematic diagram illustrating possible receptor selectivity towards G protein-dependent signalling or β-arrestin dependent signalling, resulting in various cellular responses.

Biased signalling in the chemokine system have increasingly reported by numerous studies in recent years [34–36]. Undoubtedly, biased signalling adds extra complexity to the signalling system. What causes biased signalling and what does it mean to the entire signalling system? As illustrated above, different chemokine ligands stabilise in the binding pocket of chemokine receptor in differential conformations. The differential conformational stabilisation by bound chemokines induces the activation of particular signalling pathways preferentially than others (summarised in Figure 3) [27]. Notably, some signalling pathways have been shown to be anti-tumourigenic [37–39]. In other words, targeting the selectivity towards the anti-tumourigenic pathways can potentially be a novel approach to develop targeted cancer therapy. The following sections will outline the current understanding of the mechanisms underlying biased signalling in some examples of chemokine ligand–receptor pairs and propose how this can be applied to design therapeutic candidates.

## CCL19 and CCL21/CCR7

Ectopic expression of CCR7 is implicated in tumour growth and metastasis. As reviewed by A. Salem et al. [40], there is a correlation of high expression of CCR7 and lymph node metastasis in various cancers such as breast, pancreatic and lung cancers. CCL19 and CCL21 are well-known cognate ligands of CCR7. Many studies have demonstrated the functional diversity of both ligands [34,41,42]. CCL19 induces chemotaxis via G protein-independent signalling through β-arrestins, whereas CCL21 impairs chemotaxis and yet induce ERK activation via G protein-dependent signalling [34,42].

In terms of the structural basis, the distinct 37 amino acids long, positively charged C-terminal tail of CCL21 differentiates its functional role from CCL19. CCL21 possesses a strong binding affinity to glycosaminoglycans (GAGs) due to the positively charged tail [42]. The GAG-bound CCL21 obscures its interaction with CCR7, which in turn potentially impairs chemotaxis. Evidence has shown that GAG interactions with chemokines link to the regulation of chemotaxis [43,44]. Owing to the absence of the long C-terminal tail in CCL19, CCL19 binds GAGs weakly without affecting chemotactic signalling through β-arrestins [42]. Taking into account the latest structural information on ligand–receptor interactions as mentioned above, the long C-terminal tail of CCL21 particularly interacts with polysialic acid at the N-terminus of CCR7, and subsequently induces ERK signalling via G protein [42].

To further elucidate the bias towards signalling through β-arrestins for CCL19, a recent finding reveal that the phosphorylation sites at the C-terminus of CCR7 induced by GRKs correlates to the intracellular functions of β-arrestins [36,42,45]. Emerging evidence supports that the phosphorylation patterns by different GRKs encode distinct functions of chemokine, known as phosphorylation barcode [36,48]. CCL19 activates GRK3 and GRK6 whereas CCL21 activates GRK6 only [36,42]. The additional phosphorylation sites by GRK3 in CCL19/CCR7 pair potentially contribute to β-arrestin dependent signalling [42].

A development of partial agonists based on CCL21/CCR7 interactions can be proposed. The tactic is to retain the inhibitory effect of chemotaxis from CCL21 while block other tumour-promoting effects sufficiently. In the future structure-activity relationship (SAR) study, receptor docking based on the binding interaction between the long C-terminal tail of CCL21 and polysialic acid of CCR7 can be performed to differentiate the structure of CCL21 from CCL19. After the development of a CCL21-specific backbone, the addition of a link to GAG-mimicking molecules can be considered to impair some CCL21-specific tumour-promoting effects.

## CXCL12 and CXCL12–CXCL4/CXCR4

High expression of CXCR4 is shown in brain, breast, pancreatic, ovarian, prostate and colon cancers, leading to metastasis [6]. CXCL12/CXCR4 is a well-studied chemokine ligand–receptor pair, which plays an important role in homeostasis and pathogenesis in malignant diseases including cancer [47–49]. Recent discoveries indicate that CXCL12 exists as monomers and homodimers in a balanced monomer-dimer equilibrium in normal conditions [50]. Disturbed monomer-dimer equilibrium was seen in cancer [51]. Dimeric CXCL12 differs from the cellular responses to monomeric CXCL12. Monomeric CXCL12 preferentially induces signalling through β-arrestins, whereas dimeric CXCL12 possesses selectivity towards G protein-dependent signalling via Gα_i_ [52]_._ In accord with the expected cellular responses from the two distinct signalling pathways, monomeric CXCL12 promotes chemotaxis and yet inhibits intracellular calcium mobilisation. As opposed to dimeric CXCL12, reduced chemotaxis and enhanced intracellular calcium mobilisation are observed. Both forms of CXCL12 induces ERK1/2 activation but it differs in duration [28,52]. Since monomeric CXCL12 activates post-endocytic signals through β-arrestins, more sustained ERK1/2 activation is resulted [52].

What makes monomeric and dimeric CXCL12 function differently? In the aspect of structural differences, the interface of dimeric CXCL12 favours binding to sulfotyrosines at the N-terminus of CXCR4 [28,52]. Similar to the interactions between CCL21 and CCR7, the enhanced binding affinity at CRS1 favours G protein-dependent signalling [52]. In the pathway through Gα_i_, inhibition of cAMP production, in turn, activates phospholipases responsible for intracellular calcium release. It has been confirmed that no chemotactic activity is seen in Gα_i_-dependent signalling [52]. In regard to monomeric CXCL12, GRK6 is involved in β-arrestin dependent signalling [53]. As mentioned above, the phosphorylation pattern induced by GRKs correlates to the functions of β-arrestins, resulting in differential cellular responses. In the case of monomeric CXCL12, intracellular calcium mobilisation is diminished while prolonged ERK1/2 activation is induced [53].

Apart from the formation of CXCL12 homodimers, a study shows that it is possible to form CXCL12–CXCL4 heterodimer. CXCL4 has been shown to be a ligand of CXCR3B promoting anti-tumourigenesis and apoptosis. Pairing to CXCL12, CXCL12-induced chemotaxis is reduced and inhibition of CXCL12-induced RNA expression for actin rearrangement is seen [54]. However, attention needs to be paid in the case of prostate cancer. A recent finding shows that CXCL4/CXCR12 pathway is critical in the differentiation of prostate cancer progenitor cells leading to tumourigenesis [55]. Therefore, future studies on chemokine signalling requires cancer type-specific analysis.

By exploiting the anti-tumourigenic properties of dimeric CXCL12 and CXCL12–CXCL4 heterodimers, strategies such as designing peptides mimicking CXCL12–CXCL4 binding interface and partial agonist based on the dimeric CXCL12 interface can be considered. It is aimed to suppress CXCL12-induced chemotaxis while not affecting the homeostatic functions of CXCL12/CXCR4 signalling.

## CXCL12/CXCR4–ACKR3

High expression of ACKR3 is found in glioblastoma brain tumours in particular [6]. As briefly mentioned, ACKR3 is structurally different from canonical chemokine receptors [19]. According to the literature, ACKR3 has been reported to be co-expressed with CXCR4 to modulate CXCR4 signalling in cancer cells [56–58]. It has been confirmed that ACKR3 signalling is biased towards β-arrestin dependent signalling [33,35,56,58]. Notably, alterations on the N-loop and N-terminus of CXCL12 do not affect ACKR3 activity and even CXCR4 antagonists act as agonist towards ACKR3, subsequently transducing signalling through β-arrestins recruitment. The observations imply that ACKR3 possess a binding pocket with high plasticity to diverse stimulations from various chemokines towards β-arrestin dependent signalling [33].

With respect to the underlying mechanisms on signalling modulation, considerable efforts have been made to characterise the interaction between CXCR4 and ACKR3 [56–58]. It was revealed that the site where ACKR3 interacts with the C-terminal tail of CXCR4 in the CXCR4–ACKR3 heterodimers is important for constitutive β-arrestins recruitment [58]. Through site-specific phosphorylation by GRK2, signalling through β-arrestins is induced and elicits various cellular responses, including sustained ERK1/2 activation, p38 MAPK activation and cell migration. In the meantime, attenuated Gα_i_-dependent signalling is observed [58].

As described previously, Gα_i_-dependent signalling functions partially tumour-inhibitory effects. The attenuation of Gα_i_-dependent signalling by ACKR3 is somewhat not ideal for cancer treatment. Recent studies have discovered an allosteric modulator ITAC [58] and a competitive ACKR3 antagonist ACT-1004-1239 that potentially block CXCL12-induced β-arrestin recruitment [59]. Reduced cell migration is seen in the treatment of ITAC as a result of the resumption of Gα_i_-dependent signalling [58].

## GAG interactions with chemokines — a potential target to regulate chemotaxis

Latest studies provide new insights into the role of GAGs in the regulation of chemotaxis [43,44]. As the example of CCL21/CCR7 described above, some chemokines possess a strong binding affinity to GAGs that occludes the chemokine binding to their cognate receptors, as a result, G protein-dependent signalling contributing to chemotaxis is affected [42]. Other than CCL21, ELR-CXC chemokines, such as CXCL1 [60], CXCL5 [61], CXCL7 [62] and CXCL8 [63], also bind GAGs strongly.

Intriguingly, the pattern of how the chemokine dimers bind GAG can affect chemokine receptor activation. Provided that the chemokine dimer is sandwiched between two GAG molecules, no receptor activation occurs as a result of the occlusion of the residues of chemokine responsible for receptor binding [64,65]. On the other hand, in the case that the chemokine dimer is bound to a single GAG alone, receptor activation still occurs and chemotaxis is induced due to the availability of the second binding site of the chemokine dimer for receptor interactions [66].

The findings related to GAG binding have opened a new avenue in the design of partial agonists particularly for ELR-CXC chemokines. By linking GAGs to the chemokine-mimicking backbone, partial agonists can be developed that blocks chemotaxis while maintaining other essential functions of the chemokines [64–66].

## Dynamic interactions of cholesterol and chemokine receptors — a potential target to modulate chemokine receptor functions

A new prospect on the modulatory roles of cholesterol on chemokine receptor function has been a trend in recent research [67,68]. The structural roles of phospholipid bilayer in plasma membrane have been thoroughly studied [65]. As chemokine receptors are embedded within the phospholipid bilayer in cell surface expression [17], questions have been raised whether membrane lipids interfere with the conformational integrity of chemokine receptors affecting receptor functionality.

Recent studies have been attempting to alter cellular cholesterol levels to investigate any changes in chemokine receptor cell surface expression and functioning. Interestingly, it was demonstrated that moderately reducing cellular cholesterol level increases the cell surface expression of CCR7 oligomers [69]. Enhanced expression of CCR7 oligomers promotes cell migration via Src in G protein-dependent signalling. However, further reducing cholesterol level alters the conformational integrity of CCR7, negatively impacting Src-dependent signalling in chemotaxis [69].

Based on the current findings, cellular cholesterol is potentially a target to modulate chemokine receptor expression and functioning. Reposition of cholesterol-regulating medicines, for example, statins, can be applied in cancer targeted therapy. Yet, there are still uncertainties unanswered related to the link between cholesterol and chemokine receptors. For example, are there any differences in the roles of cholesterol on different chemokine receptors and other classes of cell surface receptors? Is the effect cell-type-specific? What is the optimal level of cholesterol depletion to achieve the anti-tumourigenic effects?

## Potential applications and approaches to translate experimental findings to clinical settings

As research on chemokines as new targets for cancer therapy has become promising, emerging clinical trials have been attempting to include chemokine receptor inhibitors to optimise the efficacy of conventional chemotherapy.

A well-known anti-HIV drug, Maraviroc, also known as a CCR5 antagonist, has recently tested in the clinical course of colorectal cancer (CRC). A study revealed that influx of T cells mediated by CCL5/CCR5 contributes to CRC liver metastases [70]. Inhibition of CCR5 leads to macrophage repolarisation, in turn mitigating tumour-promoting inflammation and preventing from liver metastases of CRC [70]. The clinical study showed the potential for combination therapy to apply Maraviroc to improve the efficacy of chemotherapy in advanced CRC.

Another clinical study focused on the well-studied CXCR4 and investigated its clinical roles in pancreatic cancer. The study demonstrated that co-targeting of CXCR4 and hedgehog pathways improves the outcome of pancreatic cancer resistant to gemcitabine [71]. The findings provide an insight into a combination use of CXCR4 antagonist and hedgehog inhibitor as an adjuvant to gemcitabine, in order to maximise chemotherapy efficacy in pancreatic cancer.

Apart from targeting to chemokine receptors directly, targeting specific chemokine ligand/receptor conformations and downstream protein–protein interactions in biased signalling are alternative approaches for targeted cancer therapy. These approaches can potentially resolve the immunosuppressive effects of chemokine receptor antagonists due to generalised inhibition of chemokine signalling involved in host immune responses.

In terms of drug design to optimise targeted therapy, the utilisation of macrocyclic scaffold has increasingly been applied in drug development particularly for small molecular inhibitors. The features of orally bioavailability and membrane permeability explains that macrocycles are ideal candidate which enhances oral bioavailability in the pharmacokinetic aspect. Additionally, macrocyclic therapeutics increase selectivity to target-specific conformations [72]. Another significant advantage is adding modulating functions whilst retaining binding affinity to the target [72]. For example, a macrocycle-based drug class, antascomicins, suppresses mTOR signalling despite retaining its binding to FKBP12 [73]. As mentioned above about specific ligand–receptor conformations and biased signalling, the design of drugs targeting chemokine signalling can benefit from the unique features of macrocycles.

Another approach in drug design is the use of antibody-drug conjugate which is specific for a tumour-associated antigen. A new clinically approved drug, Mogamulizumab, is an anti-CCR4 monoclonal antibody [10]. This implies that antibody-drug conjugate can be widely used, particularly in the delivery of cytotoxic drugs to cancer tissues with minimal effects to host cells leading to immunosuppression.

## Perspectives

- Chemokine signalling plays an important role in chemotaxis, cell survival and proliferation contributing to cancer metastasis and immune diseases.
- The complexity of the chemokine system can be exploited to precisely fine-tune cellular responses via the dynamic ligand–receptor interactions and the interplay of downstream signalling proteins.
- There is much more work on mechanistical studies to define cancer type-specific chemokine signalling paradigm for the development of cancer targeted therapy.

## Open Access

Open access for this article was enabled by the participation of University of East Anglia in an all-inclusive *Read & Publish* pilot with Portland Press and the Biochemical Society under a transformative agreement with JISC.

## Abbreviations

ACKRs: atypical chemokine receptors
CRC: colorectal cancer
CRS1: chemokine recognition site 1
GAGs: glycosaminoglycans
GPCR: G protein-coupled receptor
ICL: intracellular loops
